# Cardiac function, myocardial fat deposition, and lipid profile in young smokers: a cross-sectional study

**DOI:** 10.3389/fcvm.2023.1225621

**Published:** 2023-11-14

**Authors:** Ana Natália Ribeiro Batista, Thaís Garcia, Robson Prudente, Maurício F. Barbosa, Pamela Modesto, Estefânia Franco, Irma de Godoy, Sergio Paiva, Paula Azevedo, Suzana Erico Tanni

**Affiliations:** ^1^ Pneumology Area, Department of Internal Medicine, Botucatu School of Medicine, São Paulo State University (UNESP), Botucatu, Brazil; ^2^ Pulmonary Function Laboratory, Clinical Hospital of Botucatu Medical School, São Paulo State University (UNESP), Botucatu, Brazil; ^3^ Department of Tropical Diseases and Diagnostic Imaging, Botucatu School of Medicine, São Paulo State University (UNESP), Botucatu, Brazil

**Keywords:** smoking, lipids, heart function tests, proton magnetic resonance spectroscopy, myocardial fat deposition

## Abstract

**Background:**

There is a possibility that cardiac morphometric characteristics are associated with the lipid profile, that is, the composition and concentration of triglycerides, total cholesterol, HDL, LDL, and others lipoproteins in young smokers without comorbidities. Thus, this study aimed to evaluate the association of cardiac morphometric characteristics, myocardial fat deposition, and smoking cessation with the lipid profile of young smokers.

**Methods:**

A clinical and laboratory evaluation of lipids and the smoking status was performed on 57 individuals, including both a smoker group and a control group. Cardiac magnetic resonance imaging (MRI) with proton spectroscopy was performed to identify cardiac changes and triglyceride (TG) deposition in myocardial tissue.

**Results:**

No differences were observed between the groups (control vs. smokers) in relation to the amount of myocardial TG deposition (*p* = 0.47); however, when TG deposition was correlated with cardiac MRI variables, a positive correlation was identified between smoking history and myocardial TG deposition [hazard ratio (95% CI), 0.07 (0.03–0.12); *p* = 0.002]. Furthermore, it was observed that the smoking group had lower high-density lipoprotein cholesterol [51 (45.5–59.5) mg/dl vs. 43 (36–49.5) mg/dl, *p* = 0.003] and higher TG [73 (58–110) mg/dl vs. 122 (73.5–133) mg/dl, *p* = 0.01] and very-low-density lipoprotein cholesterol [14.6 (11.6–22.2) mg/dl vs. 24.4 (14.7–26.6) mg/dl, *p* = 0.01] values. In the control and smoking groups, a negative correlation between TGs and the diameter of the aortic root lumen and positive correlation with the thickness of the interventricular septum and end-diastolic volume (EDV) of both the right ventricle (RV) and left ventricle (LV) were noted. Moreover, in the RV, positive correlations with the end-systolic volume (ESV) index (ESVI), stroke volume (SV), ESV, and EDV were observed. Regarding serum free fatty acids, we found a negative correlation between their values and the diameter of the lumen of the ascending aortic vessel. Lipoprotein lipase showed a positive correlation with the SV index of the RV and negative correlation with the diameter of the lumen of the ascending aortic vessel.

**Conclusion:**

Several associations were observed regarding cardiac morphometric characteristics, myocardial fat deposition, and smoking cessation with the lipid profile of young smokers.

## 1. Introduction

Smoking is the leading cause of preventable death worldwide, which is the main risk factor for the development of several comorbidities. It corresponds to one of the most important health problems globally, causing dependence and reaching different ages and social classes (1, 2).

It is estimated that every year, more than eight million people die due to tobacco use and approximately 1.2 million of these deaths result from passive exposure to smoking.(1) Thus, passive exposure, occasional smoking, and/or consumption of a few cigarettes a day is considered sufficient to be related to the risk of heart disease (3). On the other hand, withdrawal from tobacco exposure reduces the risk of cardiovascular events by 50% after 1 year of abstinence.(2).

In this context, tobacco is recognized as the primary isolated risk factor for acute myocardial infarction (AMI) and plays a significant role in the onset and progression of coronary artery disease (1, 4). This is due, in part, to chronic exposure to nicotine, which leads to vascular endothelial dysfunction. This dysfunction is characterized by diminished nitric oxide synthesis, vasoconstriction, and increased adhesion of leukocytes to the endothelium, contributing to the development of atherosclerosis. This process is further exacerbated by the composition and concentration of lipoproteins, including triglycerides, total cholesterol, HDL, LDL, and other lipoproteins (lipid profile), as well as abnormal plasma lipoproteins. Notably, there is a decrease in high-density lipoprotein (HDL) levels, while levels of very-low-density lipoprotein (VLDL) and triglycerides (TG) increase (5–8).

In addition, pathophysiological mechanisms are involved in the increase in cardiovascular risk. There is evidence for a direct toxic effect of cigarette smoke on the myocardium, culminating in cardiac remodeling.(9, 10) A previous experimental study showed that cigarette smoke was associated with eccentric cardiac hypertrophy, regardless of its hemodynamic effects. In addition, the activity of enzymes responsible for the oxidation of fatty acids (FA) decreased, and consequently, the cardiac TG levels increased; other findings included cell death, hypertrophy, and myocardial dysfunction (11–14).

Although smoking is directly related to the increase in the circulating free FA (FFA) levels, it is not known exactly whether there are changes in glucose and FA metabolism in the myocardial tissue of smokers or there is an association between the accumulation of TG in the heart and hypertrophy and myocardial dysfunction in these individuals (15–17). We previously observed a strong association between smoking in young adults and decline in heart function, confirming that smoking can directly influence cardiac function, even without atherosclerosis or other chronic comorbidities, suggesting that other mechanisms are involved in the cardiac remodeling process, such as insulin resistance (IR) and changes in glucose metabolism (18, 19). Therefore, cardiac magnetic resonance imaging (MRI) is an important tool for identifying cardiac alterations, and this together with proton spectroscopy can evaluate TG deposition in myocardial tissue, thus favoring a more accurate assessment of cardiac function in this population (18, 20–24).

In this sense, our hypothesis is that cardiac morphometric characteristics are associated with the lipid profile and TG deposition in the myocardium of young smokers without comorbidities. This study is justified by expanding our knowledge about smoking and alterations in heart disease, aiming to enhance the understanding of the mechanisms of action of cigarettes on the cardiovascular system. Given the aforementioned, our aim was to evaluate the association between cardiac morphometric characteristics and myocardial fat deposition with lipid profiles in young smokers.

## 2. Methods

This is a cross-sectional study in which patients from the “smoking cessation outpatient clinic” at the Clinical Hospital of Botucatu Medical School were invited to participate in the study. For the control group, posters were distributed locally, and those who also showed an interest in the study were selected (approval number, 2.076.232).

The inclusion criteria for the smoking group were individuals aged >18 years with a minimum smoking history of 10 pack-years and smoking consumption of at least one cigarette per day in the month prior to the evaluation. In the control group, individuals without previous smoking and without any comorbidity were considered. The exclusion criteria for both groups were the presence of coronary or heart failure, systemic atherosclerosis, AMI, and chronic diseases such as systemic arterial hypertension; diabetes mellitus; dyslipidemia; respiratory, hepatic, renal, psychiatric diseases; and cancer of any kind. In addition, patients who did not undergo a previous echocardiographic examination (to exclude any ischemic heart disease or cardiac remodeling), had restrictions on performing cardiac MRI, and presented with alterations in pulmonary function examination were excluded.

All study participants were evaluated between 2017 and 2018 based on their clinical history and complete physical examination. Smoking history (pack-years) and current smoking status were investigated and complemented by assessing the intensity of nicotine dependence (Fagerström test) (25). Smoking status was confirmed by measuring carbon monoxide (CO) in expired air using a standardized technique with specific equipment (Micro + Smokerlyzer; Bedfont, England, UK). Values above 6.0 ppm of expired CO were considered significant for active smoking (26). For the treatment of smoking cessation, first-line drugs were administered in this study, dispensed according to medical prescriptions. Both the follow-up of these individuals and the verification of smoking cessation occurred qualitatively (direct questions), quantitatively (Fagerström test) and biological methods, especially through the analysis of carbon monoxide in exhaled breath (27, 28).

Laboratory evaluation included the analysis of complete blood count, fasting blood glucose, fasting insulin, C-reactive protein (CRP), and serum lipid profile—total (serum) cholesterol, HDL, low-density protein (LDL), VLDL, and TG. The homeostatic model assessment (HOMA) index was used to assess IR (24, 29). FAs were analyzed through conversion to their coenzyme A derivatives, and for lipoprotein lipase (LPL) analysis, assays were performed in duplicate using commercially available enzyme-linked immunosorbent assay kits.

**Figure 1:**
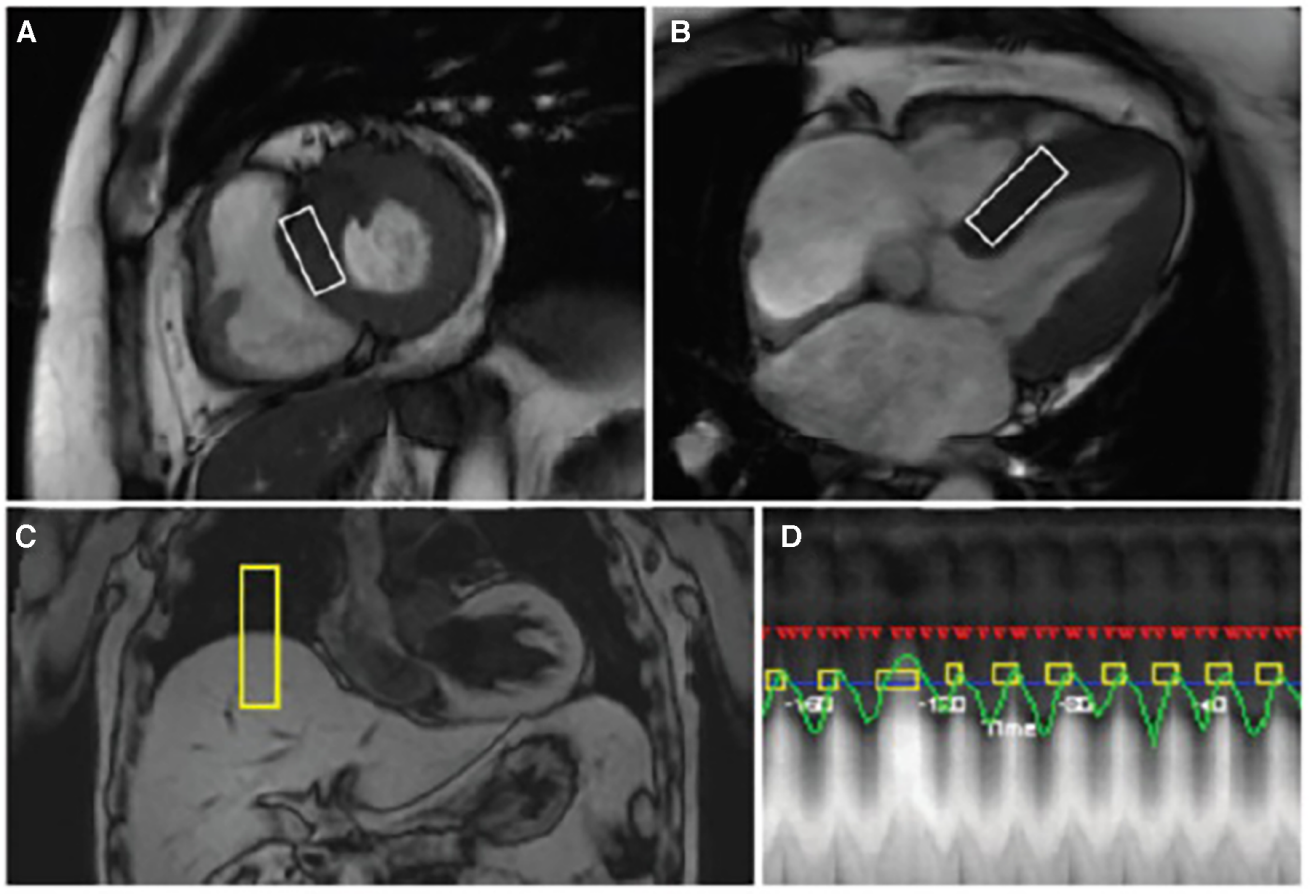
Cardiac magnetic resonance images with voxel location in the interventricular septum of the myocardium for image acquisition of a non-smoker subject. Options are: (**A**) left ventricular short axis; (**B**) four chambers; (**C**) capturing respiratory movement through the trigger located between the liver and the thorax; and (**D**) acquiring proton spectroscopy data.

All participants of the research underwent cardiac MRI examination, which were carried out in the 3-T MR device (Magnetom Verio, Siemens AG, Health care Sector, Erlangen, Germany) according to the study protocol. The localizers were obtained through image cut sequences of the heart to the programming of sequences of posterior images. Images on cine-MRI in short and long axes of the left ventricle (LV) using the Steady-State Free Precession sequence were used for calculations of ventricular volumes and functions. Proton spectroscopy was performed with voxel placement in the interventricular septum (IVS) to quantify the myocardial fat deposits (Figure 1) (30, 31). After spectroscopy, gadolinium contrast was injected (Gadolinium DTPA – 0.15 mmol/kg) and new images of late enhancement were obtained after 15 min using the phase-sensitive inversion-recovery – PSIR sequence in the short and long axes of the LV and also the T1 maps at the same anatomical plans (32–34).

Regarding the reference values of ventricular volumes for cardiac MRI, the data provided by Kawel-Boehm et al., for healthy adults of both sexes, were used.(32).

For image analysis, ventricular function, volumes and mass of the LV were calculated through the Ventricular Function Argus software (Siemens AG, Healthcare Sector). All volumes and ventricular mass were indexed to the body surface area (21). Using standardized LV segmentation, we divided the T1 maps into 16 myocardial segments for T1 time measurements independently (35). The apex (segment 17) was not analyzed because it was impossible to avoid the partial volume effect in this segment. Regions of interest (ROIs) were drawn on the pre-contrast image and copied to the postcontrast images. The extracellular volume (ECV) was calculated manually using T1 measurements before and 15 min after the administration of intravenous contrast (36). T2 measurement was performed with the ROIs positioned in the IVS to exclude the possibility that eventual increases in native T1 were due to edema.

**Figure 2:**
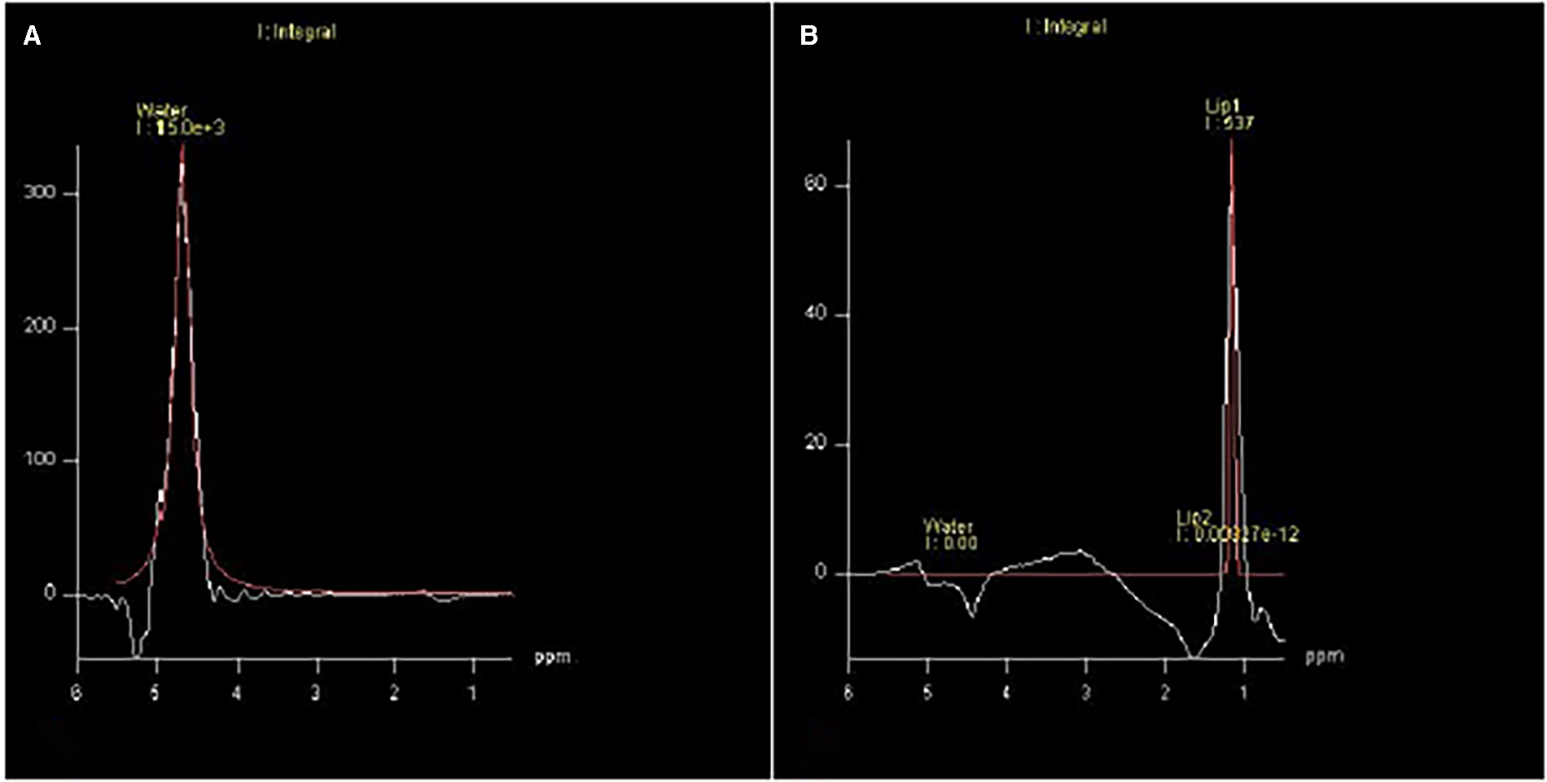
Water suppression to determine TG peaks, which were summed to obtain the TG peak value. (**A**) Without water suppression and (**B**) With water suppression.

The following formula was used to calculate the ECV: ECV = (1-Hct) × *λ*, where Hct is the hematocrit and *λ* is the gadolinium partition coefficient. To calculate spectroscopy, two simultaneous cardiac images were used (four chambers and a short axis) with placement of the voxel in the IVS for quantification. The first image was taken without water suppression for peak-water determination. The second image was obtained with water suppression to determine TG peaks, which were summed to obtain the TG peak value (Figure 2). For the final quantification of myocardial TGs, the following formula was used: Lipd1 + Lipd2/water × 100 (9). Data were analyzed using Spectroscopy Evaluation software (Siemens AG, Healthcare Sector, Erlangen, Germany).

Descriptive statistics were used to describe the characteristics of all participants. Means ± standard deviation or medians and interquartile range (25%–75%) were used depending on the data distribution. Categorical variables are expressed as percentages. The *χ*^2^ test was used to compare categorical variables.

The comparison between two independent groups was performed using Student's *t*-test or Mann–Whitney *U* test. Paired Student's *t*-test or Wilcoxon test were used to compare two dependent groups, especially before and after smoking cessation. To study the associations between the functional and morphometric variables of MRI with fat deposition, correlation coefficient analysis was performed using the Pearson or Spearman correlation test. Linear regression was used to evaluate the association of pack years, gender or age, and TG cardiac deposition.

The significance level was set at *p* < 0.05. All data were analyzed using SPSS version 17.0 (IBM Software, Dallas, TX, USA) and SAS (USA).

## 3. Results

Initially, 97 individuals of both sexes were invited to participate in this study: 47 in the control group (nonsmokers) and 50 in the active smoker group. After applying the inclusion and exclusion criteria, 28 and 29 participants remained in the control and smoking groups, respectively. Of the 29 individuals included in the smoking group, only 10 had ceased smoking at the end of the study. Table 1 shows the main characteristics of the 57 participants included in this study.

**Table 1:** Characteristics of participants.

Variables	Control (*n* = 28)	Smokers (*n* = 29)	*p*-value
Man, %	60.7	41.4	0.14^a^
Age, years	34.7 ± 4.5	36.7 ± 5.7	0.14^a^
Weight, kg	75.8 ± 12.4	71.5 ± 15.7	0.26^a^
Height, m	1.73 ± 0.11	1.65 ± 0.09	0.003^a^
BMI, kg/m^2^	25.1 ± 3.3	26.0 ± 4.0	0.39^a^
CO, ppm	2.0 (1.0–3.0)	9.5 (5.7–15.5)	<0.001^b^

Kg, kilograms; M, meters; BMI, body mass index; CO, carbon monoxide; ppm, parts per million.

Data expressed as mean ± standard deviation or median (25–75%) or percentage (*p* < 0.05).

^a^ Student's *t*-test or

^b^ Mann–Whitney.

Regarding the lipid profile, the comparison between the two groups showed a significant reduction in the HDL cholesterol levels [51 (45.5–59.5) vs. 43 (36–49.5), *p* = 0.003] and increase in the TG [73 (58–110) vs. 122 (73.5–133), *p* = 0.01] and VLDL [14.6 (11.6–22.2) vs. 24.4 (14.7–26.6), *p* = 0.01] levels in the smoking group (Table 2).

**Table 2:** Laboratory assessment.

Variables	Control (*n* = 28)	Smokers (*n* = 29)	*p*-value
Free fatty acids, mmol/L	0.42 ± 0.18	0.45 ± 0.21	0.56^a^
CRP, mg/L	0.6 (0.5–0.7)	0.6 (0.5–1.1)	0.77^b^
HOMA index, *n*	0.99 (0.50–1.44)	1.42 (0.87–2.38)	0.05^b^
Blood glucose, mg/dl	88.4 ± 20.7	85.6 ± 12.2	0.70^a^
TG, mg/dl	73 (58–110)	122 (73.5–133)	0.01^b^
Total cholesterol, mg/dl	182.5 ± 22.8	187.9 ± 31.4	0.61^a^
HDL, mg/dl	51 (45.8–59.5)	43 (36–49.5)	0.003^b^
LDL, mg/dl	114.7 ± 22.7	119.8 ± 34.7	0.71^a^
VLDL, mg/dl	14.6 (11.6–22.2)	24.4 (14.7–26.6)	0.01^b^

CRP, c-reactive protein; HOMA, homeostasis model assessment; TG, triglycerides; HDL, high-density lipoprotein; LDL, low-density lipoprotein; VLDL, very-low-density lipoprotein.

Data expressed as mean ± standard deviation or median (25–75%) or percentage (*p* < 0.05).

^a^ Student's *t*-test or.

^b^ Mann–Whitney.

When the TG deposition in the myocardium was quantified by proton spectroscopy, there was no statistically significant difference between them [0.311 (0.156–0.610) vs. 0.197 (0.116–0.572), *p* = 0.47]. As for the analysis of TG deposition in the myocardium by proton spectroscopy before and after smoking cessation, we did not observe significant differences between the initial and final moments (0.56 ± 1.08% vs. 0.16 ± 0.22%, *p* = 0.28).

Myocardial TG deposition was evaluated with cardiac MRI variables in the control and smoker groups, and there was a positive correlation between smoking history and myocardial fat deposition [coefficient (95% CI), 0.07 (0.03–0.12); *p* = 0.002]. We did not identify male influence (coefficient: 4.04; 95% CI: −6.27–14.4; *p* = 0.43; *R*^2^: 3%) or age (coefficient: 0.79; 95% CI: −0.33–1.92; *p* = 0.16; *R*^2^: 4%) in the deposition of triglycerides. No significant association was found between myocardial TG deposition and serum lipid profile variables in the control group; however, the smoking group showed a negative correlation with the LPL (Table 3). When the controls and smokers were grouped together, we did not identify a significant correlation between TG deposition and serum markers.

**Table 3:** Correlation between myocardial fat deposition and lipid profile variables for the smokers group.

	Variables	*R*	*p*-value
Triglyceride deposition	LPL, U/L	−0.38	0.04
	FFA, mmol/L	−0.07	0.72
	TC, mg/dl	0.10	0.61
	LDL, mg/dl	0.11	0.62
	HDL, mg/dl	−0.06	0.75
	VLDL, mg/dl	0.15	0.46
	TG, mg/dl	0.15	0.46
	HOMA, *n*	0.14	0.56

Correlation data between fat deposition and lipid profile variables were performed using Pearson's correlation for data with normal distribution or Spearman's correlation for non-parametric data.

LPL, lipoprotein lipase; FFA, free fatty acids; TC, total cholesterol; LDL, low-density lipoprotein; HDL, high-density lipoprotein; VLDL, very-low-density lipoprotein; TG, triglycerides; HOMA, homeostasis model assessment.

When analyzing the serum markers of all patients with cardiac MRI variables in relation to the LV, we identified a positive correlation between TG, end-diastolic volume (EDV), and IVS size. In relation to the right ventricle (RV), there was a positive correlation between the LPL and stroke volume (SV) index (SVI) and TG with EDV, end-systolic volume (ESV), SV, EDV index (EDVI), and ESV index (ESVI). Furthermore, we found a negative correlation between the ascending aorta diameter and LPL, FFAs, and TG in the aortic root (Table 4).

**Table 4:** Correlation between myocardial fat deposition and lipid profile variables for the control and smoker groups.

	Variables		*r*	*p*-value
Left ventricle	TG, mg/dl	EDV, ml	0.27	0.04
		IVS, mm	0.30	0.03
Right ventricle	LPL U/L	SVI, ml/m^2^	0.34	0.01
	TG, mG/dl	EDV, ml	0.30	0.03
		ESV, ml	0.29	0.03
		SV, ml/m^2^	0.28	0.04
		EDVI, ml/m^2^	0.28	0.04
		ESVI, ml/m^2^	0.29	0.03
		Aortic root, mm	−0.35	0.008
	AAo, mm	LPL, U/L	−0.29	0.04
		FFA, mmol/L	−0.29	0.04

Correlation data between fat deposition and lipid profile variables were performed using Pearson's correlation for data with normal distribution or Spearman's correlation for non-parametric data.

TG, triglycerides; LPL, lipoprotein lipase; AAo, ascending aorta; EDV, end-diastolic volume; IVS, interventricular septum; SVI, stroke volume index; ESV, end-systolic volume; SV, stroke volume; EDVI, end-diastolic volume index; ESVI, end-systolic volume index; FFA, fat free acids.

**Figure 3:**
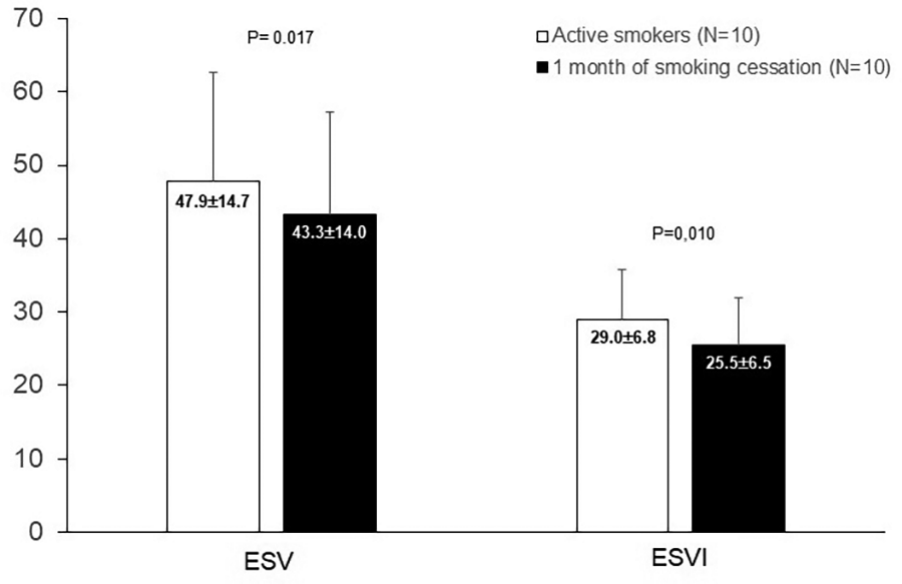
Comparison of functional variables of right ventricular of smokers at baseline and after one month of smoking cessation.

When comparing before and after 1 month of smoking cessation, in relation to the analysis of RV morphometric and functional variables, we identified a statistically significant difference only in the ESV (47.9 ± 14.7 vs. 43.3 ± 14.0, *p* = 0.017) and ESVI (29.0 ± 6.8 vs. 25.5 ± 6.5, *p* = 0.010) (Figure 3). In the LV, we identified a statistically significant difference only in the end-systolic diameter (ESD) (32.2 ± 4.6 vs. 33.8 ± 4.9, *p* = 0.03).

## 4. Discussion

In the present study we did not find strong associations between the variables analyzed and myocardial fat deposition, however, the smoking group had lower HDL and TG levels. These findings are well established in the literature, likewise the TG values and their influence on smokers have also been discussed (37–40). By itself, abdominal visceral fat accumulation is strongly correlated with higher TG levels, lower HDL values, and IR (41–43). In this sense, Freeman et al. (37) reported that smoking had a small impact on TGs of active smokers, and more recently, Koda et al. (41) demonstrated that in smokers with a greater area of abdominal visceral fat, the TG values were also higher. However, in those with less visceral fat deposition, their numbers did not differ statistically between smokers and nonsmokers.

In the cardiac MRI analysis, we found a negative correlation between the TG values and diameter of the aortic root lumen and positive correlation between the thickness of the IVS and EDV of both the RV and LV; in the RV, positive correlations with the following volumes were also noted: ESV, SV, EDVI, and ESVI. Regarding FFA, we observed a negative correlation between its values and the diameter of the vessel lumen of the ascending aorta.

Fat deposition in the cardiac tissue and adjacent structures can generate mechanical overload with consequent remodeling of the cardiac mass, alteration of vascular resistance, and ejection fraction, causing a decrease in LV performance due to eccentric changes in the ventricular chamber, systolic changes, and increased ventricular wall tension (33, 35, 40–42, 44, 45).

Notably, the greater the fat deposition in certain anatomical structures, the greater the predisposition to the emergence and development of associated dysfunctions (46). Thus, we observed several cardiovascular structural and functional alterations correlated with both TG and FFA values, and presumably, such findings may be useful in the early identification of the risk of systemic arterial hypertension, atherosclerosis, and heart failure, among others directly or indirectly related to the cardiovascular system.

Regarding IR, although below the reference value, the HOMA index was also higher in smokers, but the difference was not statistically significant. We speculate that the sample size may have hampered the analysis; however, the highest absolute values in smokers were in agreement with other studies on the subject (42, 47). IR can be a predictor of arterial hypertension and dyslipidemia, which, in turn, favor fat deposition in the cardiovascular system; however, in our sample, in the MRI analysis, we did not find correlations between the HOMA index and cardiac structures and functions (19).

Few studies have evaluated myocardial fat deposition and its clinical associations, especially with IR (48, 49). Iacobellis and Leonetti (49), using transthoracic echocardiography, demonstrated a good correlation between IR and pericardial lipid droplet accumulation. Silva et al. (45), on the other hand, found through necropsies that fat deposition in the LV is associated with risk factors for cardiovascular diseases, such as smoking and atherosclerotic disease.

Thus, on the lipid profile and IR, we observed that our results are in accordance with the literature, and in this sense, it can be seen that there may be a tendency to reduce fat deposition as the LPL activity increases, or vice versa, given that in the MRI analysis, we found that LPL presented a positive correlation with the SVI and negative correlation with the diameter of the vessel lumen of the ascending aorta (37, 40, 47).

In the comparison of LPL between controls and smokers, there was no statistically significant differences. For some years now, LPL has been investigated in smokers because smoking can induce a reduction in LPL activity in both adipose and muscle tissues, either by reducing TG hydrolysis and clearance or through hyperinsulinemia, which reduces TG hydrolysis and leads to an increase in its values in smokers (50). Thus, it can be observed that LPL may be associated with low weight in these individuals and with mass gain after smoking cessation (10, 25, 51).

However, LPL activity in smokers remains controversial (25, 52, 53). In general, LPL is the main enzyme that hydrolyzes circulating TG and releases FFA, which can be used as energy by the myocardium. Classic studies have already suggested that the greater FFA response in patients with AMI could be the result of a greater release of catecholamines after stimulation by nicotine; however, studies that have evaluated the lipid profile and its relationship with cardiac tissue are still scarce (54).

Myocardial fat is commonly seen on computed tomography and cardiac MRI in healthy adults or those with heart disease (55). The physiological and/or pathophysiological role of this fat deposition is still poorly understood; however, it may be related to conductivity changes resulting from increased oxidative stress and inflammation (42, 56).

The TG deposition determined by proton spectroscopy between smoking and control group was not significant and, in this sense, we did not find previous studies comparing TG deposition by spectroscopy specifically in smokers. However, in healthy individuals, a study by Van der Meer et al. (36) with 20 nonsmokers identified values of 0.4% ± 0.02%; Liu et al. (57) presented median values of 0.5 (0.3%–1.0%) in a sample of 92 individuals, of whom 62% were smokers; and Sai et al. (58) found mean values of 0.85% ± 0.40% in 37 participants, with no information on the number of smokers. When comparing our findings with the literature, we observed that in a sample with 50% smokers, the median TG value was 0.24 (0.12%–0.55%), a lower percentage than those found by the authors mentioned earlier.

In our sample, no significant differences were observed in individuals before and after smoking cessation in the TG deposition in the myocardium by proton spectroscopy; however, there was a reduction in the percentage values, showing a greater tendency of TG deposition in smokers than in nonsmokers, which corroborates the pathophysiology of tobacco-induced myocardial lipid peroxidation. Furthermore, it was identified that TG deposition was associated with smoking history and, only 1 month after smoking cessation, a reduction in its values was observed (59). Thus, we can speculate that the cessation time was not sufficient to detect a greater reduction in the TG values or that the power of the present study was not sufficient for this analysis.

Regarding the analysis of morphometric and functional variables after 1 month of smoking cessation, we identified only a reduction in the ESV and ESVI of the RV and a significant increase in the ESD of the LV. In relation to these findings, we can assume that some pathophysiological aspects may be associated. For example, the diastolic and systolic volumes refer to the resulting volume within the cavity at end diastole and end systole, respectively, and characteristically, we could interpret the following findings as a consequence of reduced activation of β-adrenergic receptors in the heart and renin–angiotensin–aldosterone system, which would reduce preload by reducing volume retention. Thus, the efficiency of the right cavity identified in this study would be better after smoking cessation, with a consequent reduction in the ESV and ESVI.

As for the changes in the LV, our hypothesis was that in our sample, the smoker's heart would be working adaptively without many morphological changes that we could have identified, but after smoking cessation, this previous adaptation would have become more evident. This finding may be related to the Frank–Starling mechanism, in which the preload determines the force of contraction, that is, when there is an increase in myocardial fiber distension, both the tension generated and the contractility increase (60, 61). Our findings suggest that patients who smoke had adequate preload and systolic function before smoking cessation, and after smoking cessation, the change in volume would have been evident and consequently highlighted the adaptation of the geometry by increasing the LV ESD, which may have been associated with concentric remodeling.

Comparatively, in this context, we can identify clinical studies only with patients with different characteristics, including different comorbidities; nonetheless, these were analyzed using adjusted statistical models, which can be interpreted and partially compared with our findings. The Multi-Ethnic Study of Atherosclerosis (MESA), with more than 6,000 participants with cardiac MRI assessment for atherosclerosis risk factors, showed that the group of never-smokers had better cardiac ejection fraction and lower EDVI and LVMI; however, we cannot say that ex-smokers also have the same characteristics (62). Thus, residual myocardial changes may occur after smoking cessation, as in the study by Rosen et al. who found slight changes in LV function after smoking cessation (63).

Regarding inflammation, in this study, evaluated by CRP, no statistical differences were observed between the groups of smokers and nonsmokers. In general, smokers have higher CRP values than individuals who have never smoked (64); however, as CRP is an indicator of acute inflammation, caution should be exercised when interpreting the available data on CRP levels in patients with chronic conditions or without exacerbation of CRP underlying disease, especially when excluding patients with recent infections and/or inflammation (65).

Thus, our results confirm the findings in the literature on the lipid profile and IR of smokers; however, their associations with myocardial fat deposition, assessed by MRI, were weak (10, 37–39, 66, 67). In addition to the sample size, we can speculate that physiologically, the human heart contains fat deposits that may vary in different individuals. For example, the amount of adipose tissue and its range in muscle tissue can be influenced by advancing age; there is more fat deposition in men than in women and more prominently in white people, Asians, black people, and Hispanics (33, 68). Another well-discussed aspect is that myocardial fat deposition is better observed in individuals with cardiopathies, such as healed myocardial infarction, arrhythmogenic RV dysplasia, cardiac lipoma, cardiomyopathy with muscular dystrophy, hypertrophic cardiomyopathy, and dilated cardiomyopathy (45, 49, 68, 69).

This study has some limitations that need to be reinforced. First, there is a need for further studies to confirm our findings because the sample size was small and was composed only of young smokers. Second, we did not confirm the nicotine levels to assess the impact of smoking on possible mechanisms involved. Third, this was a cross-sectional study that cannot assert causality. Fourth, there was no follow-up of smokers to assess the evolution of cardiac functions and long-term outcomes.

## 5. Conclusion

Active smoking has a direct influence on cardiac morphometric characteristics and myocardial fat deposition. Cardiac function and lipid profile can be modified early after smoking cessation in young smokers.

## Data Availability

The original contributions presented in the study are included in the article/supplementary material, further inquiries can be directed to the corresponding author.
